# Morphine Reduces Myocardial Infarct Size via Heat Shock Protein 90 in Rodents

**DOI:** 10.1155/2015/129612

**Published:** 2015-08-27

**Authors:** Bryce A. Small, Yao Lu, Anna K. Hsu, Garrett J. Gross, Eric R. Gross

**Affiliations:** ^1^Department of Anesthesiology, Perioperative and Pain Medicine, School of Medicine, Stanford University, Stanford, CA 94305, USA; ^2^Department of Pharmacology, Medical College of Wisconsin, Milwaukee, WI 53226, USA

## Abstract

Opioids reduce injury from myocardial ischemia-reperfusion in humans. In experimental models, this mechanism involves GSK3*β* inhibition. HSP90 regulates mitochondrial protein import, with GSK3*β* inhibition increasing HSP90 mitochondrial content. Therefore, we determined whether morphine-induced cardioprotection is mediated by HSP90 and if the protective effect is downstream of GSK3*β* inhibition. Male Sprague-Dawley rats, aged 8–10 weeks, were subjected to an *in vivo* myocardial ischemia-reperfusion injury protocol involving 30 minutes of ischemia followed by 2 hours of reperfusion. Hemodynamics were continually monitored and myocardial infarct size determined. Rats received morphine (0.3 mg/kg), the GSK3*β* inhibitor, SB216763 (0.6 mg/kg), or saline, 10 minutes prior to ischemia. Some rats received selective HSP90 inhibitors, radicicol (0.3 mg/kg), or deoxyspergualin (DSG, 0.6 mg/kg) alone or 5 minutes prior to morphine or SB216763. Morphine reduced myocardial infarct size when compared to control (42 ± 2% versus 60 ± 1%). This protection was abolished by prior treatment of radicicol or DSG (59 ± 1%, 56 ± 2%). GSK3*β* inhibition also reduced myocardial infarct size (41 ± 2%) with HSP90 inhibition by radicicol or DSG partially inhibiting SB216763-induced infarct size reduction (54 ± 3%, 47 ± 1%, resp.). These data suggest that opioid-induced cardioprotection is mediated by HSP90. Part of this protection afforded by HSP90 is downstream of GSK3*β*, potentially via the HSP-TOM mitochondrial import pathway.

## 1. Introduction

Strategies for mitigating organ damage from ischemia-reperfusion injury during surgical procedures are a continued topic of research for anesthesiologists and surgeons. In particular, an experimental model of cardiac ischemia-reperfusion injury is frequently used to study agents, interventions, and molecular mechanisms which reduce myocardial damage. The translation of these models into the operating room, particularly during cardiovascular surgeries such as valve replacements and coronary artery bypass grafting, has produced encouraging strategies to reduce the associated reperfusion injury when an aortic cross-clamp is released, such as ischemic preconditioning, remote conditioning, volatile anesthetics, and opioids.

Interestingly, the clinical improvement observed with remote conditioning is enhanced by concomitantly administering morphine to patients [1]. Volatile anesthetics [2], ischemic preconditioning [3], and remote conditioning [4] all involve an opioid receptor-dependent mechanism. Studying experimentally how opioids reduce myocardial injury may provide cellular targets to design drugs to mitigate organ ischemia-reperfusion injury and determine which cellular targets are important to allow protection from ischemia-reperfusion injury. Prior studies we performed were the first to characterize in rodents the cellular mechanism of opioid-induced cardioprotection, involving *δ*-opioid receptors [5] and a central role for glycogen synthase kinase 3*β* (GSK3*β*) inhibition [6].

The mitochondria are important cellular organelles regulating cardioprotection. The import of proteins including protein kinase B (AKT) [7], protein kinase C (PKC) [8], and connexin 43 [9] into the mitochondria to promote cardioprotection requires heat shock protein 90 (HSP90) for cellular import by the translocase of mitochondrial membrane (TOM) complex. Formation of this complex relies on the conserved C-terminus EEVD sequence motif of HSP90 and its protein partner heat shock protein 70 (HSP70, also known as heat shock cognate protein 71). This EEVD sequence is a tetratricopeptide repeat domain (TPR), which interacts with the TOM70 allowing transfer of proteins from the HSP complex to TOM70 [10, 11].

Inhibition of the HSP90 ATP domain by geldanamycin reduced the functional recovery afforded by GSK3*β* inhibition [12]. In addition, GSK3*β* inhibition also changed the protein mitochondrial content, including increasing HSP90*β* and HSP70 [12]. This previous finding suggests that GSK3*β* could regulate mitochondrial import of proteins through the HSP-TOM complex. Further, whether the TPR domain is important for cardioprotection is unknown.

For this issue highlighting ischemia-reperfusion injury and anesthesia, the purpose of our study was to determine whether opioid-induced cardioprotection is mediated by HSP90. We examined the importance of the ATP and TPR domains of HSP90 and whether inhibition of the ATP or TPR domain affects infarct size reduction afforded by morphine or GSK3*β* inhibition.

## 2. Methods

Procedures and protocols were approved by the Animal Care and Use Committee at both Stanford University and Medical College of Wisconsin. All animal studies conformed to the National Institute of Health* Guide for the Care and Use of Laboratory Animals*. Initial experiments were performed at the Medical College of Wisconsin, followed by completion of these study groups after establishing an identical experimental set-up at Stanford University. Eight- to 10-week-old male Sprague-Dawley rats (Charles River) were used for the studies outlined.

### 2.1. Pharmacological Agents Used

**Figure 1:**
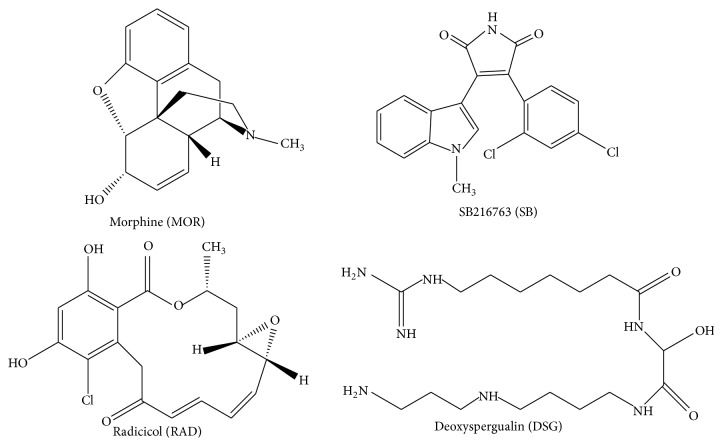
Chemical structure of agents used for the study. Morphine, the opioid-receptor agonist (top left), SB216763, the GSK3*β* inhibitor (top right), radicicol, the HSP90 ATP site inhibitor (bottom left), and deoxyspergualin, the C-terminus TPR domain inhibitor (bottom right). Abbreviations used throughout additional figures are placed in ().

The chemical structures of the drugs used are depicted (Figure 1). Morphine (Sigma, 0.3 mg/kg IV bolus) was dissolved in saline. This dose was determined from prior studies using our rodent model, which included determining the dose-dependent effects of morphine on myocardial infarct size [13]. The GSK3*β* inhibitor, SB216763 (Tocris, 0.6 mg/kg), was dissolved in DMSO (1 mg/mL, given in < 0.2 mL volume). The dose was selected based upon prior studies [6, 14]. Two HSP90 inhibitors used were radicicol (Tocris, 0.3 mg/kg) and deoxyspergualin (DSG, 0.6 mg/kg) both dissolved in DMSO (1 mg/mL, given in < 0.2 mL volume). Radicicol inhibits the ATP binding site at the N-terminus of HSP90 [5]. DSG interferes with the EEVD C-terminus sequence motif of HSP90, limiting protein interactions with the C-terminus [15, 16]. The volume of DMSO delivered does not affect myocardial function as determined from our prior studies using this model [6, 17].

### 2.2. Sequence Analysis

For heat shock protein amino acid sequences, a search was performed using the Swiss-Prot database. We scanned the sequences for the last 8 amino acids of the C-terminus for each heat shock protein family. Further, predictive protein phosphorylation of serine and threonine sites on both HSP90*α* and HSP90*β* were determined by NetPhos 2.0.

### 2.3. *In Vivo* Myocardial Infarction Rodent Model

**Figure 2:**
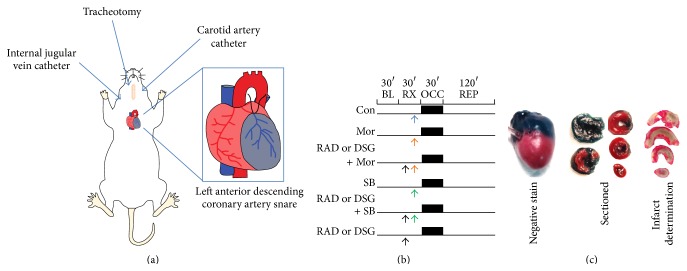
Experimental protocol description. (a) Pictorial description of the rat* in vivo* myocardial infarction protocol. Surgical preparation involved a tracheotomy, carotid cannulation to measure blood pressure, and internal jugular vein cannulation to deliver drugs. Ischemia was generated by snaring the left anterior descending coronary artery and subsequently releasing the snare for reperfusion. (b) Experimental protocol. After 30 minutes of baseline, rats were subjected to treatment and subsequently 30 minutes of ischemia followed by 2 hours of reperfusion. HSP90 inhibitors were given 5 minutes prior to morphine, SB216763, or vehicle. (c) Representative staining for infarct size. After 2 hours of reperfusion, the LAD was again occluded and the area at risk was negatively stained by patent blue dye (far left). Following, the left ventricle was sliced into five equal pieces (middle). Lastly, the tissue was stained for viable tissue which turned red, while nonviable infarcted tissue remained white (far right). BL = baseline, RX = drug treatment, OCC = ischemia, and REP = reperfusion. Blue arrow = saline, orange arrow = morphine, green arrow = SB, and black arrow = RAD or DSG.

The model was previously described in a number of publications [13, 14]. Briefly, rats were anesthetized with Inactin (thiobutabarbital, 100 mg/kg intraperitoneal). After obtaining body weight, a tracheotomy was performed in addition to cannulation of the carotid artery and internal jugular vein to measure blood pressure and administer drugs, respectively (Figure 2(a)). Rats were placed on a ventilator (30–40 breaths per minute, tidal volume 8-9 mL/kg) and adjusted to maintain a normal pH and end tidal CO_2_ by a blood gas machine (Radiometer ABL80). Body temperature was maintained at 37-38°C by heating pads and heat lamps. The heart was exposed by an incision in the fourth-intercostal space, the pericardium was excised, and a suture was placed around the left anterior descending coronary artery (6-0 Prolene, Ethicon). After surgical manipulation and adjustment of ventilator setting based upon blood gas analysis, rodents were allowed to stabilize for 30 minutes prior to initiation of the experimental protocol.

The experimental protocol consisted of nine treatment groups (Figure 2(b)). Rats received either saline, morphine, or the GSK3*β* inhibitor, SB216763, 10 minutes prior to ischemia. In select groups, the HSP90 inhibitors, radicicol or DSG, were given 5 minutes prior to this treatment. Subsequently, rats were subjected to 30 minutes of left anterior descending (LAD) coronary artery occlusion followed by 2 hours of reperfusion. Two hours after reperfusion, the LAD was again occluded and the heart negatively stained for the area at risk by injection of patent blue dye (Sigma) given through the internal jugular vein. Following, the heart was then excised, both the atria and the right ventricle were removed, and the left ventricle was sliced into 5 equal slices to create cross sections from apex to base. The slices were separated into normal zone and area at risk, followed by incubation in 1% TTC to measure viability of myocardial tissue (Figure 2(c)). Viable tissue stained red, while nonviable tissue remained unstained or white. Infarct size as a percentage of area at risk was determined gravimetrically. Heart rate, blood pressure, and rate pressure product were monitored and calculated throughout the experimental protocol.

### 2.4. Statistical Analysis

All groups are reported as mean ± SEM. A one-way ANOVA followed by Bonferroni correction for multiplicity was used in order to compare each group to the control group. This test was used to determine statistical significance for infarct size, area at risk, and hemodynamic parameters. Statistical analysis was performed using GraphPad Prism 6.

## 3. Results

**Figure 3:**
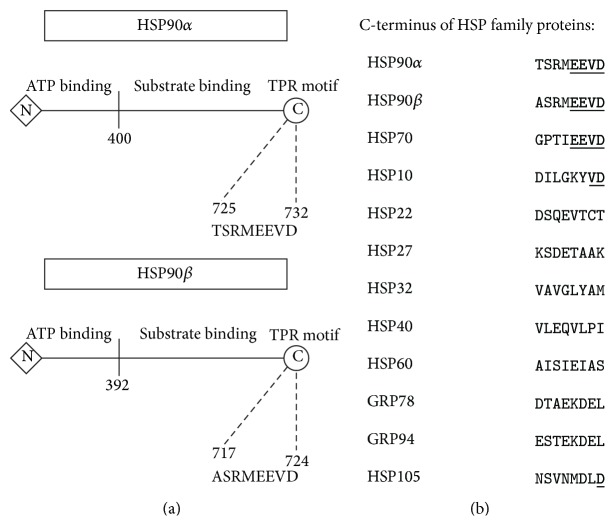
Structure of HSP90*α* and HSP90*β*. (a) General schematic of HSP90*α* and HSP90*β*, each having an N-terminus ATP site, substrate site, and C-terminus TPR motif. (b) Presence of C-terminus EEVD motif for both HSP90 and HSP70 in humans (underlined). Other human HSP family sequences lacked the C-terminus motif. Sequences obtained from the Swiss-Prot database: P07900 HS90_HUMAN, P08238 HS90B_HUMAN, P08107 HSP71_HUMAN, P61604 CH10_HUMAN, Q9UJY1 HSPB8_HUMAN, P04792 HSPB1_HUMAN, P09601 HMOX1_HUMAN, P25685 DNJB1_HUMAN, Q0VDF9 HSP7E_HUMAN, P11021 GRP78_HUMAN, P14625 ENPL_HUMAN, and Q92598 HS105_HUMAN.

Both HSP90*α* and HSP90*β* are comprised of an ATP domain, substrate domain, and a TPR motif (Figure 3(a)). Since the initial report that deoxyspergualin inhibits HSP70 and HSP90 by the EEVD C-terminus TPR sequence motif was made 17 years ago [15], we first scanned the Swiss-Prot database of protein sequences for heat shock proteins looking for additional heat shock proteins with an EEVD sequence motif. Much to our surprise, the EEVD C-terminus sequence motif only existed for HSP90*α*, HSP90*β*, and HSP70 (Figure 3(b)) and was highly evolutionary conserved. This suggests that DSG is a selective antagonist which will block the EEVD motif on HSP90 and HSP70.

We then tested our hypothesis in an* in vivo* rat model of myocardial ischemia-reperfusion injury. 65 animals were used to obtain 60 successful experiments. Five rats were excluded, 3 due to anesthetic overdose prior to group assignment and 2 rats due to intractable ventricular fibrillation during reperfusion (control group and radicicol + SB216763 group). Hemodynamics including heart rate, blood pressure, and rate pressure product had no differences in baseline values when groups were compared to control. The radicicol + SB216763 group mean arterial pressure was significantly different during ischemia compared to the control group. Furthermore, the rate pressure product for the morphine group was significantly different compared to the control group at reperfusion (Table 1).

**Figure 4:**
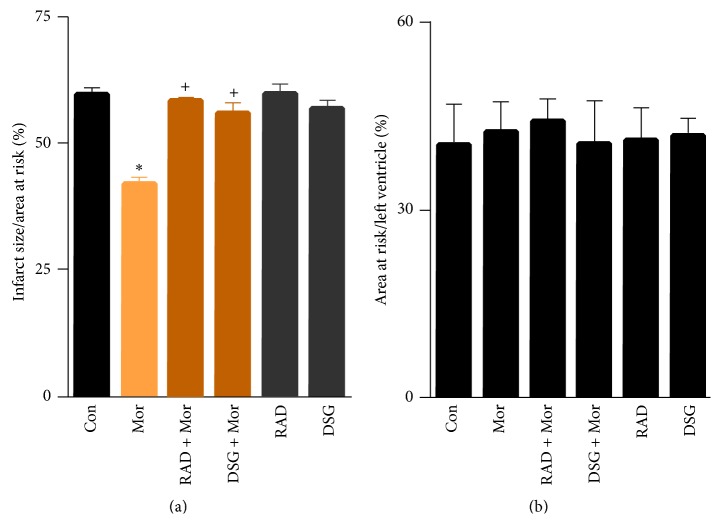
Infarct size and area at risk results for morphine experiments. (a) Infarct size/area at risk%. Morphine (Mor) significantly reduced myocardial infarct size compared to control. Either radicicol (RAD) or deoxyspergualin (DSG) blocked morphine-induced infarct size reduction. (b) Area at risk/left ventricle for each group. Data presented as mean ± SEM; ^*∗*^ *P* < 0.01 versus control, ^+^ *P* < 0.01 versus morphine.

Initially, we tested whether inhibition of HSP90 blocks opioid-induced cardioprotection. Morphine reduced myocardial infarct size significantly compared to control (Figure 4(a), 42 ± 2^*∗*^% versus 60 ± 1%, ^*∗*^ *P* < 0.01). When either radicicol or DSG was administered prior to morphine, the infarct size sparing effect of morphine was abrogated (Figure 4(a), 59 ± 1%, 56 ± 2%, resp.). No further increase in infarct size was noted for either radicicol or DSG given alone (Figure 4(a), 59 ± 1%, 56 ± 2%, resp.). No differences were observed for the area at risk ratio to left ventricle (Figure 4(b)).

**Figure 5:**
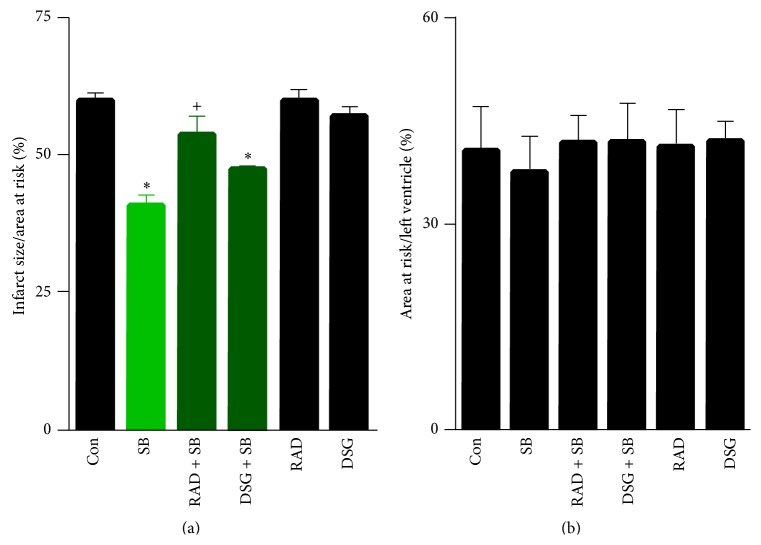
Infarct size and area at risk results for SB216763 experiments. (a) Infarct size/area at risk%. SB216763 (SB) significantly reduced myocardial infarct size compared to control. Either radicicol (RAD) or deoxyspergualin (DSG) partially blocked SB216763-induced infarct size reduction. (b) Area at risk/left ventricle for each group. Data presented as mean ± SEM; ^*∗*^ *P* < 0.01 versus control, ^+^ *P* < 0.01 versus SB216763. Control, RAD, and DSG groups were reproduced from Figure 4 for comparison purposes.

We also tested whether inhibition of HSP90 blocks the cardioprotective effect of GSK3*β* inhibition. GSK3*β* inhibition by SB216763 reduced myocardial infarct size (Figure 5(a), 41 ± 2%). The effect on myocardial infarct size reduction was approximately equal to that of morphine. In the presence of either radicicol or DSG, only partial inhibition of the infarct size sparing effect was noted (Figure 5(a), 54 ± 3%, 47 ± 1%). No differences were also noted in the ratio of area at risk to left ventricle (Figure 5(b)).

**Figure 6:**
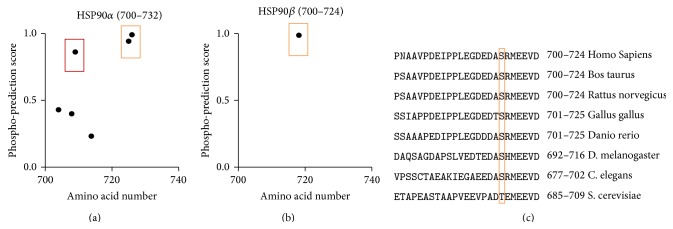
Computational analysis of HSP90*α* and HSP90*β* C-terminus. (a) HSP90*α* and (b) HSP90*β* prediction of Ser or Thr phosphorylation probability, using NetPhos 2.0. (c) Conservation of HSP90*β* EEVD sequence motif at C-terminus, including a serine, 7 amino acids from the C-terminus. In particular, this EEVD sequence is strongly conserved throughout evolution, in addition to the phosphorylation site near the C-terminus (orange box). Sequences obtained from the Swiss-Prot database: P08238 HS90B_HUMAN, Q76LV1 HS90B_BOVIN, P34058 HSPB_RAT, Q04619 HS90B_CHICK, O57521 HS90B_DANRE, O02192 HSP83_DROAV, Q18688 HSP90_CAEEL, and P02829 HSP82_YEAST.

Then we evaluated the probability for phosphorylation of either serine or threonine amino acids within the C-terminus for HSP90. This revealed high probability phosphorylation sites for threonine and serine by NetPhos 2.0 near the C-terminus for HSP90*α* in humans (Figure 6(a)). HSP90*β*, found to be increased after GSK3*β* inhibition in the mitochondria in a previous study [12], had only one potential phosphorylation site near the C-terminus (Figure 6(b)). Interestingly, the serine and EEVD portion of HSP90*β* was evolutionarily conserved in all organisms from* Homo Sapiens* to* C. elegans* (Figure 6(c)). Even* S. cerevisiae* had an equal substitution of Thr for Ser at this specific site.

## 4. Discussion

This study describes a role for both the ATP and the TPR domains of HSP90 in mediating opioid-induced cardioprotection. In a previously published study, pharmacological inhibition of GSK3*β* altered the mitochondrial content of proteins compared to untreated hearts, including statistically significant elevations of HSP90 and HSP70. The functional recovery afforded by GSK3*β* inhibition in isolated hearts for this study was also blocked in the presence of geldanamycin [12]. Our study extends these findings and further suggests that cardioprotection by GSK3*β* inhibition is abrogated by blocking either the TPR or the ATP domains of HSP90. DSG may also be a unique tool to assist in studying mitochondrial protein import due to inhibiting the EEVD motif of HSP90 and HSP70 [15].

The phosphorylation of the serine site and EEVD motif on HSP90 are closely evolutionarily conserved for all species containing mitochondria. The EEVD sequence on HSP90 and HSP70 is essential for interacting with the TPR domain of TOM70 for the mitochondrial import of proteins [10, 11]. When the C-terminus serine of HSP90 is phosphorylated, this preferentially targets HSP90 for ubiquitination and proteolysis by 3-fold when compared to the nonphosphorylated form [17]. GSK3*β* may directly regulate the mitochondrial protein import complex through C-terminus phosphorylation of HSP90. This is supported by our data showing that the efficacy of GSK3*β* inhibition-induced cardioprotection is partially blocked by selectively inhibiting the C-terminus EEVD motif of HSP90 by DSG. An* in vitro* activity assay also showed that the Ser at the C-terminus of the HSP90 can be phosphorylated by GSK3*β* in the presence of ATP (see Figure 6 for sequence) [17]. In this manner, when GSK3*β* is active, it may phosphorylate the HSP90 C-terminus and promote degradation. However, when GSK3*β* is inhibited, the complex may preferentially promote import into the mitochondria by preferential binding to the TOM70 TPR domain. This was supported by studies performed in an isolated heart model in which GSK3*β* inhibition by SB216763 increased mitochondrial protein content of both HSP90*β* and HSP70 compared to control [12].

What is unexplained is how GSK3*β* phosphorylates HSP90* in vitro* since the studies were performed without a priming kinase [17]. HSP90*β* lacks S/T-X-X-X-S/T motif at the serine closest to the C-terminus region. Less common than interactions with primed motifs, GSK3*β* can interact with unprimed sequence motifs including for proteins tau and presenilin-1 [18, 19]. It is considered that regions rich in proline, such as the region of HSP90, are more likely to contain unprimed motifs for GSK3*β* interaction.

Computationally, the probability of phosphorylation was quite high at the C-terminus region. One must consider that glutamic acid mimics a phosphorylated serine or threonine site due to its negative charge. Potentially, this may suggest that HSP90 may not require a priming protein for phosphorylation at this site, since the HSP90 motif would be SRMEEVD (S-X-X-X-E), with the Glu acting as a phosphorylated primed mimetic for GSK3*β*. This is particularly interesting since the sequence is highly conserved throughout evolution (Figure 6). Another study suggests that an interaction exists between GSK3*β* and HSP90 by coimmunoprecipitation [20]. However, this needs further investigation to confirm that this is valid in cardiomyocytes.

A number of potential limitations should be noted for this study, leading to further questions to be addressed for this signaling pathway in the future. In particular, we did not show that morphine phosphorylated GSK3*β* at Ser^9^ through biochemical assays. However, this previously was extensively studied and supported from our prior studies [6, 21]. We also only examined one time point of morphine-induced cardioprotection with morphine given prior to ischemia. From prior studies, we know that morphine also reduces infarct size equally when given either prior to or during ischemia [13]. Furthermore, the window of protection for morphine and other opioids, including methadone and *δ*-selective opioid agonists, was previously characterized [13, 22]. DSG is also not a specific HSP90 inhibitor since it will also inhibit HSP70 at the C-terminus. However, our study does suggest similar results with a specific inhibitor of the ATP domain binding site of HSP90. Further, the selectivity of DSG for HSP90 and HSP70 may be a valuable tool to study mitochondria import through the HSP90-HSP70 complex and TOM70. Lastly, we did not perform a series of vehicle-treated DMSO controls for our study, since we have previously shown that DMSO given at the present volumes will not affect infarct size in our prior studies [6, 21].

Chemotherapeutics targeting HSP90 are presently being tested in clinical trials. Interestingly, our findings suggest if HSP90 is blocked, pathways of protection from ischemia-reperfusion injury will be mitigated, regardless of whether the ATP or TPR binding sites of HSP90 are targeted. Potentially, whether HSP90 targeted chemotherapeutics affect the cardiovascular system, including mitochondrial integrity, will need further investigation. This is not unprecedented since doxorubicin, a chemotherapeutic given to treat cancers such as leukemia, lymphoma, or neuroblastoma, is known to cause cardiovascular injury and may progressively lead to heart failure.

Clinically, opioid-induced cardioprotection is reported for studies including adults and children [23–25]. Small clinical studies using either remifentanil or morphine as a treatment arm described beneficial effects in reducing myocardial injury during adult coronary artery bypass surgery [23, 24]. Further, the beneficial effects of remote conditioning can also be enhanced by providing morphine just prior to balloon inflation during a percutaneous coronary intervention [1]. These findings support a role for opioids in reducing cardiac injury during ischemia-reperfusion in humans. However, the lethal side-effects of opioid administration including respiratory depression and addiction suggest limitations of using opioids as long-term cardioprotective agents. Uncovering the molecular mechanisms involved in opioid-induced cardioprotection can lead to designing therapeutics without the deleterious side effects.

## 5. Conclusions

**Figure 7:**
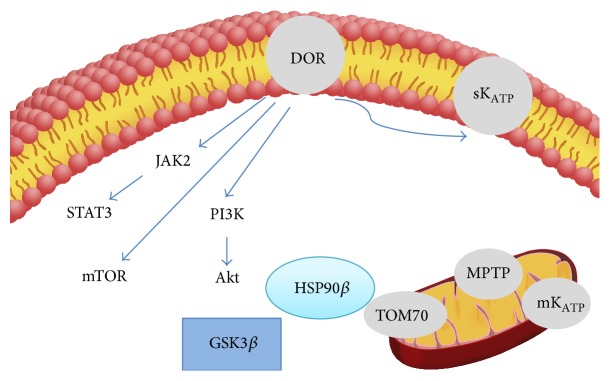
Summary schematic of opioid-induced cardioprotection. Opioids reduce myocardial infarct size through *δ*-opioid receptors (DOR) and sarcolemmal K_ATP_ channels. Subsequent activation of phosphoinositol-3 kinase (PI3k), protein kinase B (AKT), target of rapamycin (mTOR), JAK2, and STAT3 occur upstream of GSK3*β* inhibition. From this study, HSP90 is important for regulating opioid-induced cardioprotection, partially downstream of GSK3*β* inhibition. GSK3*β* likely regulates mitochondrial import through the HSP90 complex with TOM70.

The opioid-induced pathway leading to myocardial salvage is continuing to be characterized in detail. Our findings from this study suggest the importance of HSP90 in regulating opioid-induced cardioprotection, where this HSP90 effect is partially mediated downstream of GSK3*β* inhibition (Figure 7). The interaction between GSK3*β* and the HSP-mediated mitochondrial import of proteins as a general mechanism for cardioprotection needs further study.

## Figures and Tables

**Table 1:** Hemodynamics during experimental protocol.

	*n*	Baseline			15 min ischemia			2 hr reperfusion		
		HR	MAP	RPP	HR	MAP	RPP	HR	MAP	RPP
Vehicle	7	375 ± 20	121 ± 5	51 ± 2	367 ± 20	106 ± 9	41 ± 2	366 ± 18	78 ± 8	36 ± 2
MOR	7	382 ± 23	121 ± 6	47 ± 3	378 ± 23	111 ± 7	42 ± 5	338 ± 35	65 ± 4	27 ± 2^*∗*^
RAD + MOR	7	343 ± 9	111 ± 5	45 ± 2	304 ± 13	74 ± 5	27 ± 2	311 ± 11	66 ± 4	28 ± 2
DSG + MOR	7	346 ± 9	127 ± 3	50 ± 3	347 ± 7	117 ± 6	45 ± 2	296 ± 9	82 ± 7	30 ± 2
SB21	7	401 ± 8	108 ± 7	46 ± 4	395 ± 11	106 ± 10	44 ± 5	360 ± 12	81 ± 4	32 ± 2
RAD + SB	7	376 ± 11	107 ± 7	45 ± 3	363 ± 12	69 ± 5^*∗*^	28 ± 2	371 ± 13	72 ± 4	33 ± 2
DSG + SB	5	384 ± 8	124 ± 4	56 ± 2	384 ± 6	107 ± 7	45 ± 3	380 ± 8	79 ± 3	37 ± 1
RAD	7	361 ± 12	125 ± 5	52 ± 2	355 ± 10	129 ± 5	50 ± 2	333 ± 10	77 ± 4	33 ± 2
DSG	6	392 ± 7	110 ± 4	50 ± 1	388 ± 5	94 ± 10	42 ± 3	375 ± 7	78 ± 6	36 ± 3

Heart rate (HR), mean blood pressure (MAP), and rate pressure product (RPP), assessed at baseline, during ischemia and at 2 hours of reperfusion. Data presented as mean ± SEM. ^*∗*^ *P* < 0.01 versus control.
